# Changes in the level of fetoplacental complex hormones in pregnant women with miscarriage

**DOI:** 10.25122/jml-2021-0089

**Published:** 2021

**Authors:** Kateryna Mykolaivna Lisova, Iryna Valentynivna Kalinovska, Svitlana Hryhorivna Pryimak, Petro Yuriyovych Tokar, Valentin Nicolae Varlas

**Affiliations:** 1. Department of Obstetrics and Gynecology, Bukovinian State Medical University, Chernivtsi, Ukraine; 2. Department of Obstetrics and Gynaecology, Carol Davila University of Medicine and Pharmacy, Bucharest, Romania

**Keywords:** miscarriage, hormones, placenta, estradiol, placental lactogen, chorionic gonadotropin, progesterone, placental dysfunction, trophoblast, ATP – adenosine triphosphate, FPC – fetoplacental complex, hCG – human chorionic gonadotropin, PL – placental lactogen.

## Abstract

The purpose of the study was TO analyze the fetoplacental complex hormone levels and changes in their dynamics in pregnant women with miscarriage and the impact of these features on the subsequent course of pregnancy. Hormone levels were determined at different stages of gestation in 50 healthy women with a physiological course of pregnancy (control group) and 50 pregnant women with a history of miscarriage (main group). The women of the main group had a significantly slower rate of increase in hormones and a lag in quantitative indicators than the control group. The estradiol level indicators were 4.1 times (76.0%) and 2.89 times (65.5%) lower in women with miscarriage in the embryonic and late fetal period, respectively, compared to healthy women. Indicators of the level of placental lactogen and chorionic gonadotropin in the embryonic period in women with miscarriage were lower by 39.1% and 50.9%, respectively, compared to healthy women. In the late fetal period, the level of these hormones was lower by 72.9% and 35.4%, respectively. In the embryonic and late fetal periods, progesterone levels were lower by 67.4% and 68.4%, respectively, compared to the control group. The data obtained are evidence of a pronounced hormonal abnormality of the placenta, and hence a marker of fetoplacental dysfunction, which on the background of miscarriage develops at the early stages and continues to progress with the course of pregnancy.

## Introduction

Early pregnancy disorders are quite common, accounting for 10–20% of the total number of pregnancies [1]. The average prevalence of women with a single miscarriage in her medical history is 11% [2, 3]. This significantly affects the physical and psychological well-being of women. Studies show that the stress level associated with miscarriage can be equivalent to the stillbirth rate of a premature baby and can cause post-traumatic stress disorder [4, 5]. A recent meta-analysis showed that the risk of miscarriage increases by a consistent biological gradient from 11% in women who have no history of miscarriage to 64% in women with a history of six or more previous miscarriages [6, 7].

The function of the fetoplacental complex (FPC), which synthesizes several hormones and proteins, plays an essential role in ensuring the normal course of pregnancy [8]. Disorders of the hormonal function of the fetoplacental complex, which is assessed by changes in the level of hormones produced by it, underlie the pathogenesis of various complications of pregnancy, including miscarriage [9, 10].

Progesterone and human chorionic gonadotropin (hCG) are the most common serum markers used to assess the viability of pregnancy when ultrasound data are ineffective [11, 12]. Progesterone, which is produced by the corpus luteum, is vital for maintaining early pregnancy. HCG from the villous trophoblast supports the production of lutein progesterone and promotes the onset of progesterone and estradiol production by the placenta at about 8–9 weeks of pregnancy [13]. In order to be able to assess the development of pregnancy, it is important to know not the absolute value of the hormone concentration, which is determined at a certain period of pregnancy, but the change in its concentration (increase or decrease) with the course of pregnancy. In order to be able to assess the functional state of the fetoplacental complex better, it is necessary to simultaneously determine the levels of several produced hormones in dynamics [14, 15].

The aim of the study was to analyze the fetoplacental complex hormone levels and changes in their dynamics in pregnant women with miscarriage and the impact of these features on the subsequent course of pregnancy.

## Material and Methods

During pregnancy, we determined the content of the following hormones: estradiol, chronic gonadotropin, placental lactogen, and progesterone. The studies were carried out on 50 pregnant women with miscarriage (main group). The control group consisted of 50 pregnant women with uncomplicated pregnancies. Patients were examined at the Chernivtsi Regional Perinatal Center during 2020–2021.

Determination of hormones was carried out by solid-phase enzyme-linked immunosorbent assay (ELISA). The following sets of reagents were used: “Steroid ELISA-progesterone-01”, “Placental lactogen ELISA”, “Gonadotropin ELISA-HCG-1”, “Estradiol ELISA”. The studies were performed on an enzyme-linked immunosorbent assay according to the instructions for these sets of reagents.

## Results

Analysis of changes in estradiol levels in the studied groups of pregnant women (Table 1) showed significant differences. As can be seen from the results of the study, pregnant women with miscarriage during the entire gestation process (from embryonic to the late fetal period) have significantly lower estradiol levels than pregnant women in the control group. Thus, the estradiol concentration in the main group in the embryonic period was lower by 76.0% compared to the control group. In the early fetal period, the level of estradiol in the blood of pregnant women with miscarriage was lower by 86.7% compared to the control group. In the late fetal period, estradiol levels remained low, on average by 65.5% lower compared to the control group.

**Table 1.** The level of estradiol (nmol/l) in the serum of pregnant women from the main and control groups.

**Groups**	**Gestational periods**			
	**Embryonic**	**Early-fetal**	**Medium-fetal**	**Late-fetal**
**Main group**	0.93±0.06	1.82±0.38	18.50±1.19	21.38±1.57
**Control group**	3.86±0.18	13.86±0.46	43.85±3.72	61.95±7.23

The difference is reliable for pregnant women in the control group (p <0.05).

When analyzing the dynamics of changes in the levels of estradiol in the blood of pregnant women, it was found that in the embryonic period, at the early stages of the formation of the placental complex, there is a significant decrease in the estradiol level in pregnant women against the background of miscarriage. This, in turn, led to the manifestation of clinical signs of early placental dysfunction in pregnant women of the main group in the form of a risk of abortion, spotting, chorionic separation at the early period, and spontaneous miscarriage. The lowest level of estradiol was found in women with dead pregnancies, and it was on average 0.28±0.01 nmol/l.

In the late fetal period, the estradiol level in the serum of pregnant women of the main group was 21.38±1.57 nmol/l, which is 2.89 times (65.5%) lower than the control group (61.95±7.23 nmol/l). Estradiol deficiency during this period is due to a gradual decrease in the compensatory capacity of the placenta and the progression of placental insufficiency. Indicators of hCG and placental lactogen (PL) levels in the blood of pregnant women are shown in Table 2.

**Table 2.** The level of human chorionic gonadotropin, placental lactogen (mg/l) in pregnant women with miscarriage (M±m).

**Groups**	**Gestational periods**							
	**Embryonic**		**Early-fetal**		**Medium-fetal**		**Late-fetal**	
	**PL**	**HCG**	**PL**	**HCG**	**PL**	**HCG**	**PL**	**HCG**
**Main group**	0.078±0.004	14125±1107.0	1.020±0.1180	13720±1024.0	1.315±0.2380	9900±1305.0	4.045±0.654	15133±1125.0
**Control group**	0.128±0.0170	52100±2137.0	1.320±0.1860	17325±1120.0	3.414±0.2380	17370±1250.0	8.236±0.887	23430±1035.0

The difference is reliable for pregnant women in the control group (p <0.05). HCG - human chorionic gonadotropin; PL - placental lactogen.

Analyzing the hormonal status of pregnant women in the main group, we found that the content of chorionic gonadotropin and placental lactogen in the blood plasma of pregnant women throughout the gestation period was significantly lower compared to the control group.

The obtained indicators of placental lactogen content, which tended to decrease throughout pregnancy, confirm its effect on fetal weight and regulation of lipid, protein, and carbohydrate metabolism of the fetus. Thus, in the embryonic period, the level of PL in the main group was 1.6 times lower (39.1%) than in the control group. A decrease in the PL level leads to a slowdown in metabolic processes in the maternal body and impairs the transport of the placental barrier. This, in turn, is manifested by miscarriages and stillbirths in the early stages. Subsequently, a low level of PL leads to a lag in fetal weight from the gestational age, that is, to the fetal growth retardation syndrome. The most pronounced differences in the PL levels of pregnant women with miscarriage were observed in the late fetal period (72.9% lower), which indicates a progressive decompensation of the placenta function.

In the early stages, hCG is synthesized in the epithelium of the villi of the syncytiotrophoblast, and its transport is oriented towards the intervillous lacuna – into the uteroplacental blood flow. This hormone influences the development and formation of chorionic villi, the branching of the choral tree. When studying the hCG level in pregnant women of the main group, a decrease in its concentration was observed during the entire gestational process.

The rate of increase in hormone levels in the blood of women in the main group was lower compared to the control group. In the embryonic period, the hCG level in women of the main group was 3.6 times lower (by 72.9%) than in the control group; in the early and middle fetal periods, the levels were lower by 20.8% and 43.0% compared with controls; in the late fetal period, it was lower by 1.5 times (35.4%).

From the data obtained, it can be seen that the most pronounced decrease in the level of hCG was observed in the early stages of pregnancy. As a result, in pregnant women with miscarriage, the first wave of trophoblast invasion is disturbed. At the same time, there are no gestational changes in the spiral arteries; rheological changes occur in the intervillous lacuna, the so-called afunctional zones and pseudo-infarctions are formed. All this leads to a sharp narrowing of the lumen of the vessels and their complete obliteration. The consequence of this is an inadequate blood supply to the placenta, which leads to further progression of placental insufficiency.

We also determined the level of progesterone in the blood of pregnant women. The obtained changes in the level of progesterone in the serum of pregnant women are shown in Table 3.

**Table 3.** The level of progesterone (nmol/l) in the serum of pregnant women (M±m).

**Groups**	**Gestational periods**			
	**Embryonic**	**Early-fetal**	**Medium-fetal**	**Late-fetal**
**Main group**	35.5±2.1	46.7±4.61	127.4±7.42	135.0±2.0
**Control group**	108.9±2.81	157.1±23.86	288.0±38.05	427.2±62.5

The difference is reliable for pregnant women in the control group (p <0.05).

According to the analysis results, it was found that progesterone level in the blood of pregnant women in the main group was lower compared to the control group. So, in the embryonic period, the indicators of the control group were 3.1 times (67.4%) higher than those of the main group. A similar trend is observed throughout the gestational period. In the early and middle fetal periods, the progesterone level in women with miscarriage was 70.2% and 55.7% lower, respectively, compared to healthy pregnant women. In the late fetal period, the difference in indicators was 68.4% (3.2 times lower than normal). As can be seen from the obtained indicators, the progesterone level, the main hormone that contributes to the preservation of pregnancy in women with miscarriage, was significantly lower than normal during the entire gestational process. This is evidence of progressive placental dysfunction.

## Discussion

In pregnant women, estrogens are the product of a single FPC because the placenta produces them from precursors synthesized in the fetus. The intensity of biosynthesis and the amount of estrogen formed is determined by the condition of the adrenal glands of the fetus and depends on the number of androgenic precursors entering the placenta [16–18]. As a result, estrogen levels characterize not only the functional state of the placenta but also the condition of the fetus.

The deficiency of estrogen for up to 8 weeks inhibits the synthesis and reduces the activity of enzyme systems, inhibits energy metabolism, the accumulation of glycogen and adenosine triphosphate (ATP), and increases the contractile activity of the uterus [17, 19]. The most pronounced imbalance in the level of estradiol in the blood of pregnant women with miscarriage was observed in the early fetal period during the formation of the placenta, where it was 86.7% lower than the control level. This is due to the peculiarities of placenta morphogenesis. There is a violation of the first wave of cytotrophoblast invasion and, as a result, incomplete gestational remodeling of the segments of the spiral arteries. The walls of the vessels are not completely replaced by the fibrinoid, and the formed placental vessels do not provide a constant flow of arterial blood into the intervillous lacuna. As a result, the uterine-placenta site and the formed placenta are unable to meet the needs of the developing fetus. The placenta does not entirely fulfill the endocrine function [20].

Chorionic gonadotropin and placental lactogen (PL) produced by placental syncytiotrophoblast cells were determined from the protein hormones of the placenta. With the development of placental insufficiency and inferior function of the trophoblast, the synthesis and secretion of hCG are disrupted, resulting in a decrease in its content in the blood [11]. This causes a decrease in the synthesis of the level of estrogen and progesterone in the corpus luteum of the ovary and later in the placenta [21]. A drop in the level of these hormones, which ensure the course of normal biochemical and physiological processes in the muscles of the uterus, leads to an increase in the contractile activity of the myometrium, which is manifested by the phenomena of placental insufficiency in the early embryonic and early fetal periods – spotting, threat of abortion, partial separation of the chorion [12, 22].

It is known that the main site of PL synthesis is the syncytiotrophoblast of the villi with certain participation of the cytotrophoblast in the composition of the septum, cell islets, placental bed, and amniotic membranes [8, 23]. Under the conditions of the development of placental insufficiency in pregnant women with miscarriage, there is a thinning of the cytoplasm in the syncytiotrophoblast of villi, depletion of the cytoplasm of organelles, which leads to a decrease in the endocrine function of the villi epithelium [24].

Progesterone is required for the secretory transformation of the endometrium, which allows implantation and support of pregnancy at an early stage. Progesterone promotes the hyperpolarizing action on the membranes of myometrial cells and inhibits the contractile activity of uterine muscles [25, 26]. A progesterone problem often referred to as a “luteal phase defect” is thought to be one of the causes of genetically normal euploid miscarriage. Progesterone is important for maintaining decidua, and it has been suggested that corpus luteum dysfunction may lead to low progesterone levels, which may increase the risk of miscarriage [27, 28].

It has been shown that progesterone stimulates the production of the blocking P-inducing protein 34-KDa, which prevents inflammatory reactions to the trophoblast by blocking natural killer cells. Recently, several studies have shown that deregulation of the number of natural killer cells and/or their activity in the blood and endometrium is associated with various manifestations of reproductive failure [29, 30]. Decreases in progesterone levels indicate a lack of placental steroidogenesis.

## Conclusions

Women with miscarriage had a much slower increase in hormone levels and a lag in quantitative indicators compared with healthy women. This is the evidence of placental hormonal dysfunction, and hence a marker of developing fetoplacental dysfunction. The more pronounced differences in the PL level in pregnant women with miscarriage in the late fetal period indicate a progressive decompensation of the placenta function and can lead to perinatal loss. Also, the lowest level of hCG in the early stages of pregnancy leads to morphological changes and inadequate blood supply to the placenta, which leads to further progression of placental insufficiency.

Furthermore, progesterone, the main hormone that contributes to the preservation of pregnancy in women with miscarriage, was significantly lower than normal during the entire gestational process. This is evidence of placental dysfunction, which in pregnant women with miscarriage develops in early gestation terms and progresses during pregnancy. Decreases in progesterone and estradiol levels indicate a lack of placental steroidogenesis. Estradiol showed the same deviation from the norm as progesterone, indicating a deficiency in the ovaries’ response to an increase in hCG levels. Therefore, pregnant women with a history of miscarriage are at high perinatal risk.

## Acknowledgments

### Ethical approval

The ethical approval for this study was obtained by the authors from the Ethics Committee of the Bukovinian State Medical University (approval ID: 3/16.10.2018).

### Consent to participate

Written informed consent was obtained from the participants.

## Conflict of interest

The authors declare that there is no conflict of interest.
